# Application of System Biology to Explore the Association of Neprilysin, Angiotensin-Converting Enzyme 2 (ACE2), and Carbonic Anhydrase (CA) in Pathogenesis of SARS-CoV-2

**DOI:** 10.1186/s12575-020-00124-6

**Published:** 2020-06-19

**Authors:** Reza Zolfaghari Emameh, Reza Falak, Elham Bahreini

**Affiliations:** 1 Department of Energy and Environmental Biotechnology, National Institute of Genetic Engineering and Biotechnology (NIGEB), 14965/161, Tehran, Iran; 2 Immunology Research Center, Iran University of Medical Sciences, Tehran, Iran; 3 Department of Biochemistry, Faculty of Medicine, Iran University of Medical Sciences, Tehran, Iran

**Keywords:** SARS-CoV-2, COVID-19, Angiotensin-converting enzyme 2 (ACE2), Neprilysin, Carbonic anhydrases (CAs), Renin angiotensin system (RAS), Acute respiratory syndrome, Respiratory acidosis

## Abstract

**Background:**

Coronavirus disease 2019 (COVID-19) is caused by severe acute respiratory syndrome coronavirus 2 (SARS-CoV-2) appears with common symptoms including fever, dry cough, and fatigue, as well as some less common sysmptoms such as loss of taste and smell, diarrhea, skin rashes and discoloration of fingers. COVID-19 patients may also suffer from serious symptoms including shortness of breathing, chest pressure and pain, as well as loss of daily routine habits, pointing out to a sever reduction in the quality of life. COVID-19 has afftected almost all countries, however, the United States contains the highest number of infection (> 1,595,000 cases) and deaths cases (> 95,000 deaths) in the world until May 21, 2020. Finding an influential treatment strategy against COVID-19 can be facilitated through better understanding of the virus pathogenesis and consequently interrupting the biochemical pathways that the virus may play role in human body as the current reservoir of the virus.

**Results:**

In this study, we combined system biology and bioinformatic approaches to define the role of coexpression of angiotensin-converting enzyme 2 (ACE2), neprilysin or membrane metallo-endopeptidase (MME), and carbonic anhydrases (CAs) and their association in the pathogenesis of SARS-CoV-2. The results revealed that ACE2 as the cellular attachment site of SARS-CoV-2, neprilysin, and CAs have a great contribution together in the renin angiotensin system (RAS) and consequently in pathogenesis of SARS-CoV-2 in the vital organs such as respiratory, renal, and blood circulation systems. Any disorder in neprilysin, ACE2, and CAs can lead to increase of CO_2_ concentration in blood and respiratory acidosis, induction of pulmonary edema and heart and renal failures.

**Conclusions:**

Due to the presence of ACE2-Neprilysin-CA complex in most of vital organs and as a receptor of COVID-19, it is expected that most organs are affected by SARS-CoV-2 such as inflammation and fibrosis of lungs, which may conversely affect their vital functions, temporary or permanently, sometimes leading to death. Therefore, ACE2-Neprilysin-CA complex could be the key factor of pathogenesis of SARS-CoV-2 and may provide us useful information to find better provocative and therapeutic strategies against COVID-19.

## Background

Since the emergence of severe acute respiratory syndrome coronavirus 2 (SARS-CoV-2) and the worldwide outbreak of coronavirus disease 2019 (COVID-19), the number of infected and death cases have exceeded 4,300,000 and 290,000, respectively until May 21, 2020. Taken together all the observations, world health organization (WHO) declared COVID-19 as a pandemic viral infection based on the prevalence rate and severity of the disease [1, 2] (Table 1).

**Table 1:** Statistics of worldwide COVID-19 cases until May 21, 2020

Continent	Country (top 3 countries for confirmed cases)	Confirmed cases	Deaths	Recovered cases
Asia	Iran	> 129,000	> 7250	> 100,560
	India	> 113,300	> 3450	> 45,900
	China	> 82,960	> 4600	> 78,200
Europe	Russia	> 317,500	> 3100	> 92,600
	Spain	> 279,500	> 27,900	> 197,000
	UK	> 248,300	> 35,700	N/A
North America	United States	> 1,595,000	> 95,000	> 371,000
	Canada	> 80,100	> 6000	> 40,800
	Mexico	> 56,600	> 6100	> 38,900
South America	Brazil	> 294,100	> 19,000	> 117,000
	Peru	> 104,000	> 3000	> 42,000
	Chile	> 53,600	> 544	> 22,500
Africa	South Africa	> 18,000	> 340	> 9000
	Egypt	> 14,200	> 680	> 4000
	Morocco	> 7200	> 200	> 4200
Oceania	Australia	> 7100	> 100	> 6500
	New Zealand	> 1500	> 20	> 1450
	French Polynesia	> 60	0	> 60

The COVID-19 patients suffer from several clinical symtoms including fever, dry cough, fatigue, headache, sore throat, loss of taste and smell, aches and pains, diarrhea, skin rashes and/or discoloration of fingers and toes, shortness of breathing, chest pressure and pain, and loss of speech and movement. Since the outbreak of COVID-19 around the world, many academic groups and pharmaceutical companies have focused on developing therapeutic compounds to provide an effective vaccine against COVID-19. The studies revealed that remdesivir [3], combination of lopinavir and ritonavir (weak recommendation) [4], corticosteroids [5], interferon along with combination of lopinavir and ritonavir [4], and immunoglobulin-therapy through transfusion of immune plasma can be the possible thearapetic options for treatment of COVID-19. For COVID-19 prevention, the studies can focus on the results obtained from SARS-CoV vaccine clinical trials such as application of DNA, viral vector, subunit, viral-like particle, inactivated virus, and live-attenuated virus platforms [6].

To achieve an effective treatment, it is necessary to know more about the mechanism of action and pathogenesis of SARS-CoV-2. Four structural proteins including spike (S), membrane (M), nucleocapsid (N), and envelope (E) antigens are the main constituents of SARS-CoV-2 [7]. S protein is a 150 kDa glycoprotein with a N-terminal signal peptide sequence, which is glycosylated at endoplasmic reticulum (ER) [8]. The most abundant structural protein is M protein (25–30 kDa) comprising three transmembrane domains with crucial roles in the virion formation and stabilizing the complexes during the virion assembly through binding of a small glycosylated N-terminal region of M protein to N protein [9]. N protein (50–60 kDa) binds to RNA through N-terminal domain (NTD) and C-terminal domain (CTD), which is induced by phosphorylation of N protein, genomic packaging signal (GPS), replicase-transcriptase complex (RTC), and transcription regulatory signal (TRS) [10]. E protein (8–12 kDa) is a transmembrane protein with ion channel activity and a major role in assembly and release of virions from the infected cells [11]. Although E protein is not required for replication of the virus, it it plays role in pathogenesis of SARS-CoVs.

At the cellular level, the first viral entry step is the binding of the viral trimeric S protein to the human angiotensin converting enzyme 2 (ACE2) receptor [12]. Moreover, CD147 [13], dendritic cell-specific intercellular adhesion molecule-3-grabbing non-integrin (DC-SIGN, CD209) [14] and L-SIGN (CD209L) [15] are other entry receptors for SARS-CoV-2. ACE2 is a glycosylated zinc metallopeptidase and a part of the local renin-angiotensin system (RAS). Based on native function of type I membranous endopeptidases in modification of N-terminal region of proteins, ACE2 contains an extracellular N-terminal active site [16]. This enzyme is expressed in most tissues including kidneys, heart, intestine and lungs, and hydrolyse circulating angiotensisn molecules. ACE2 counteracts the effects of ACE by converting angiotensin I into Ang-(1-7) and reducing the amount of angiotensin II and balancing the ratio of circulating Ang II/ Ang-(1-7) levels [17, 18]. Elevated Ang II production in the pulmonary system triggers local vascular permeability which may result in lung edema [19, 20]. Moreover, Ang II induces pulmonary vasoconstriction in response to hypoxia. Ang-(1-7) has vasodilator, anti-proliferative, and anti-angiogenic properties, while Ang II is a vasoconstrictor, a mitogenic, and an angiogenic factor [21].

In acute respiratory distress syndrome (ARDS), if the RAS does not help in oxygenation, the general lung injury will result in pulmonary failure. Most probably, in ARDS models, ACE2 knockout will result in more severe symptoms, while overexpression might protect the lung from further injury [22].

As mentioned, ACE2 is also exploited by SARS-CoV-2 as the entry receptor. Due to the high expression of ACE2 in the lungs, intestines, respiratory tract, and also enteric system, these organs are the main sites of SARS-CoV-2 infections that are manifested by symptoms like fever, cough, pneumonia, shortness or difficulty of breathing, anorexia, sometimes diarrhea, and abdominal pain [23]. Therefore, it is considered that coronavirus infection can be controlled by blocking ACE2 and preventing the virus from binding to the cells [12]. Despite the mentioned assumption, some animal studies have shown that an increase in Ang-(1-7) has a protective effect against virus-induced lung injury via the vasodilatory effects [22, 24]. It seems that, persistent angiotensin II activity may be partly responsible for organ injury in COVID-19 [19].

Due to insufficient information on the infectious mechanism of the virus, most studies have focused on preventing the first stage of viral infection by reducing ACE2, but ACE2 knock down leads to high activation of local RAS and increased tissue injury, hypertension and renal and cardiovascular complications. Therefore, the evaluation of the efficacy of recently designed vaccines for COVID-19 requires appropriate animal models such as ACE2-transgenic and ACE2-knockout mice [25].

Although, ACE2 is the major producer of Ang-(1-7), there are other enzymes such as neprilysin, prolyl-endo-peptidase (PEP) and prolyl-carboxy-peptidase (PCP) that can produce Ang-(1-7) from Ang I [26, 27]. Among the aforementioned enzymes, high expression levels of neprilysin have been detected in the lung, especially in pulmonary epithelial cells [21]. This zinc-dependent MME is expressed in a wide variety of tissues and in addition to hydrolyzing the Ang I to Ang-(1-7), it is involved in converting several endogenous propeptides into their functional form. According to evidence, neprilysin regulates lung tissue responses to broncho-constriction inducing peptides [21, 28]. Thus, upregulation of neprilysin can be considered as a compensatory mechanism to control the concentration of Ang-(1-7) levels during COVID-19 treatment.

Airway management and hyperbaric oxygen therapy (HBOT) is the main supportive treatments for patients with severe COVID-19 as well as severe pneumonia [29]. Previous studies have revealed that the levels of both angiotensinogen and Ang II receptors increases in hypoxic conditions [30, 31]. Consequently, hypoxic conditions in COVID-19 will increase Ang II concentration through downregulation of ACE2 [32, 33]. Lungs and alveoli as the main parts of lower respiratory system have the main role in the oxygen (O_2_) exchange with carbon dioxide (CO_2_) at the molecular level; CA plays a crucial function in this respiratory exchange [34, 35]. CA as another Zn-metalloenzyme catalyzes the hydration of CO_2_ into carbonic acid and vice versa [36–38]. One of the adaptive responses to hypoxia includes the increased expression and functional activation of CA IX [39]. In this study, we applied a system biology approach to explore the association of ACE2, neprilysin, and CA, which may ameliorate COVID-19 symptoms in the lungs alveoli.

## Methods

### Identification of Coexpression of ACE2, Neprilysin, and CA

To identify role of coexpression of ACE2, neprilysin, and CA in COVID-19 pathogenesis, we used COXPRESdb v7 (https://coxpresdb.jp/) [40]. In this database, we applied the CoexViwer algorithm to identify and draw the coexpression of genes, which are linked to activation of neprilysin. This database facilitates the visualization of the multiple gene coexpression information derived from defined animals which were examined with a variety of transcriptomics technologies.

### Identification of the Association of Neprilysin and ACE2

To identify the association of neprilysin and ACE2, we employed KEGG (Kyoto Encyclopedia of Genes and Genomes) PATHWAY database [41]. This database represents the information on the reactions, relation networks, and molecular interactions of the molecules.

### Identification of Organ Localization of ACE2, Neprilysin, and CA

To identify the organ localization of ACE2, neprilysin, and CA, we used THE HUMAN PROTEIN ATLAS online program (https://www.proteinatlas.org/) [42]. This program shows the map of the localization of human proteins in cells, organs, and tissues through application of various system biology, proteomics, and transcriptomics technologies.

## Results

### Identification of Coexpression of ACE2, Neprilysin, and CA

**Fig. 1:**
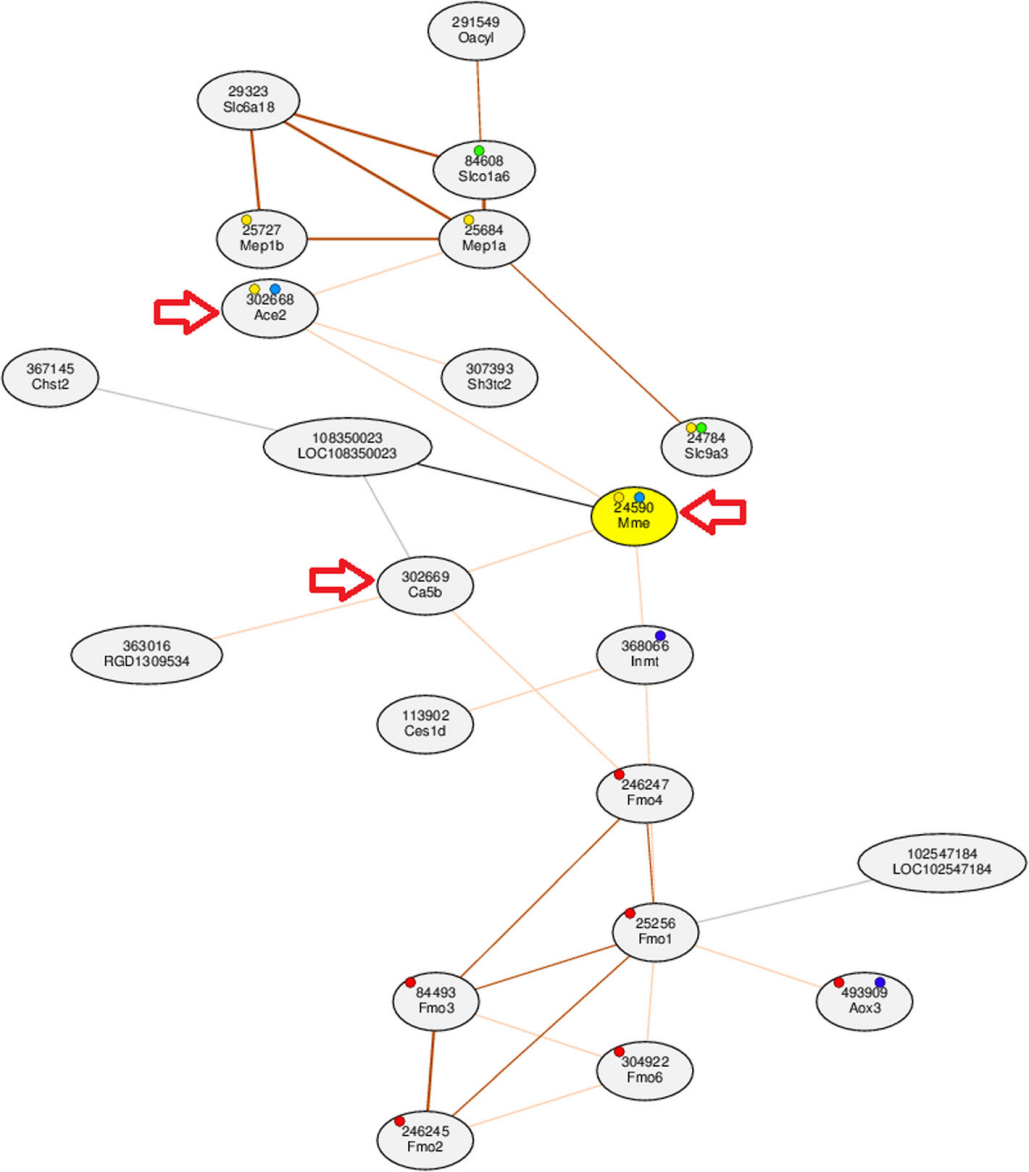
Coexpression of ACE2, neprilysin or MME, and CA in RAS. Red arrows are showing ACE2-Neprilysin-CA association network. The network consist of 22 proteins that two out of them are still not characterized

The coexpression study revealed that ACE2, neprilysin, and CA are codominantly expressed in the local RAS. The analysis showed that at least 22 genes were coexpressed in the local RAS, which can be linked to COVID-19. This association can be a direct or an indirect coexpression effect; so lack of function of a protein as the consequence of COVID-19 infection may induce the expression or activity of another protein in a different pathway (Fig. 1) (Table 2).

**Table 2:** Genes directly connected with neprilysin on the coexpression network

Locus	Function	Entrez Gene ID
CA VB	carbonic anhydrase 5B	302,669
ACE2	angiotensin-converting enzyme 2	302,668

### Identification of Association of Neprilysin and ACE2

**Fig. 2:**
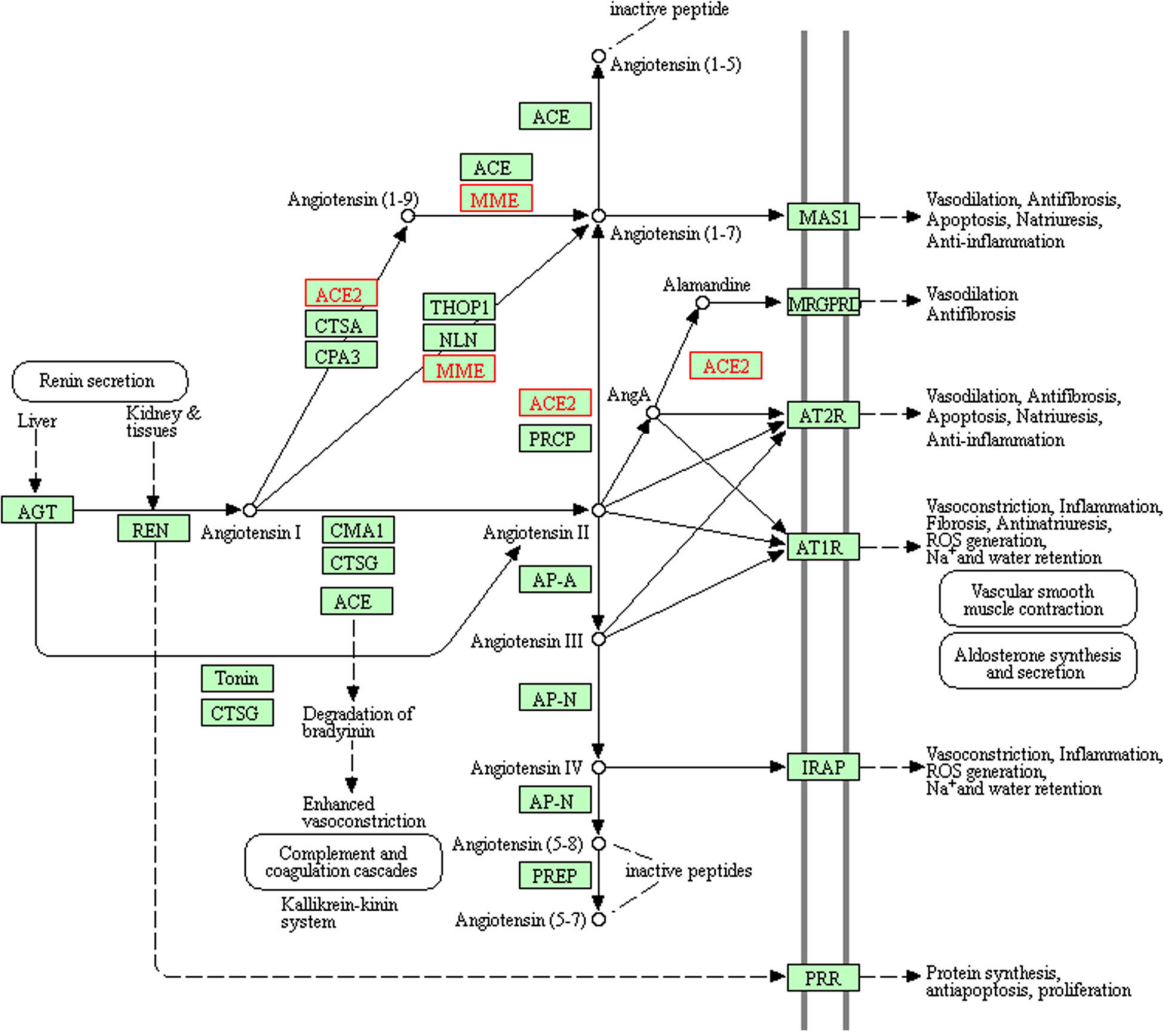
Association of neprilysin or MME and ACE2. ACE2 is crucial in conversion of angiotensin I to Ang-(1-9) angiotsnsin II to Ang-(1-7), and angiotensin A (AngA) to alamandine. In addition, neprilysin or MME is essential in conversion of angiotensin I to Ang-(1-7) and Ang-(1-9) to angiotensin Ang-(1-7)

The KEGG PATHWAY defined that neprilysin and ACE2 have association in the RAS. In this system, via two individual biochemical procedures, neprilysin converts Ang I and Ang-(1-9) to Ang-(1-7). In addition, in the RAS, ACE2 facilitates three individual biochemical procedures including conversion of Ang l to Ang-(1-9) as the substrate of neprilysin, Ang II to Ang-(1-7), and Ang A to alamandine which binds to Mas-related G protein-coupled receptor (MrgprD) to perform the vasodilatory action (Fig. 2).

### Identification of Organ Localization of ACE2, Neprilysin, and CA

**Fig. 3:**
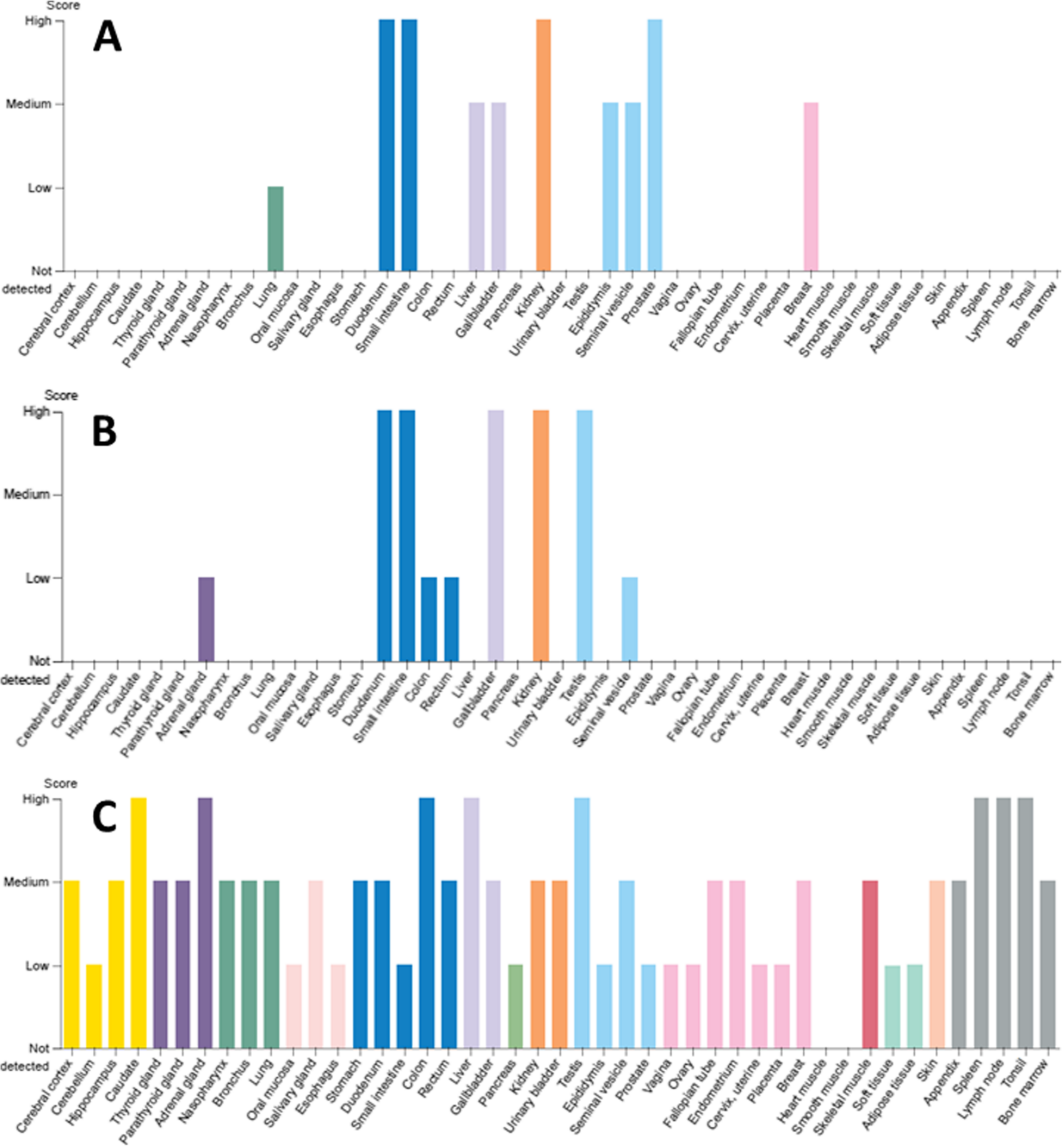
Localization of (**a**) neprilysin or MME, (**b**) ACE2, and (**c**) CA VB in the human organs. THE HUMAN PROTEIN ATLAS shows the overlap of bars and association of ACE2, neprilysin, and CA in various organs including digestive, renal, respiratory, and reproductive systems

Our analysis based on the data obtained from THE HUMAN PROTEIN ATLAS revealed that neprilysin and ACE2 are highly expressed in the digestive, renal, respiratory, and reproductive systems. The expression pattern of CA in the atlas has shown that this protein is expressed in all human organs. These overlaps in the organ localization of ACE2, neprilysin, and CA demonstrate the possible association of these proteins as a complex in COVID-19 infections through overexpression or down regulation of coding genes expression (Fig. 3).

## Discussion

The successful computational methodologies were employed previously to design viral vaccines against influenza A, hepatitis A, HIV-1, dengue, and West Nile viruses so a new era was considered in the vaccine design studies, which was named reverse vaccinology 2.0 [43–45]. This approach was designed and implemented to demonstrate the importance of combination of human immunology, clinical biochemistry, microbial genomics, and system biology to establish novel multidisciplinary approaches in the pathogenesis studies of pathogens and vaccinology researches [45, 46].

The bioinformatics analysis reveals the moderate coexpression of ACE2, neprilysin, and CA genes in the human body. As soon as SARS-CoV-2 is entered into the human oral cavity via direct contact or sneezing and cough droplets of a COVID-19 patient, the virus S protein can be attached to the ACE2, the main host cell receptor of SARS-CoV-2 expressed by epithelial cells of different sites of the oral cavity [47, 48]. Single-cell transcriptome study revealed that the expression rate of ACE2 in the tongue tissue is higher than buccal and gingival tissues [49]. Therefore, primarily SARS-CoV-2 multiplicities in the human mucosa of the oral cavity and consequently a high number of viruses are transmitted to the secondary organ targets expressing ACE2. For this reason, some have claimed drinking water could be a safe way against SARS-CoV-2 which wash the virus into the stomach where acid will inactivate it.

Single-cell sequencing (scRNA-Seq) has shown a high expression rate for ACE2 in type II alveolar (AT2) cells of lung [50], oral cavity epithelial cells [51], upper esophagus [14] and cholangiocytes [52]. A reduction in ACE2 levels due to internalizing by attached SARS-CoV-2 leads to increase in Ang II and consequently vasoconstriction in the lung [53]. Such condition causes respiratory disorders and insufficient air conditioning in alveoli which stimulates lung to upregulate ACE2 expression [54]. According to bioinformatics predictionof the coexpression of ACE2, neprilysin, and CA; an increase in neprilysin and upregulation of CA may attenuate the severity of disease and compensate the alveolar ventilation [55–59]. The elevated Ang II in lung tissue following a decrease in ACE2 by COVID-19 binding may increase the comorbidities, most likely due to inducing local and systemic inflammation. The proinflammatory effects of Ang II are mediated by increased oxidative stress. Elevated serum concentrations of C-reactive protein (CRP) is an indicator of systemic inflammation in such conditions [60].

ACE2 also is expressed in other tissues including proximal tubular cells of kidney [61], colon and ileum of the digestive system [62], urothelial cells of the gallbladder [49], and liver, pancreas and heart myocytes [63], which can justify COVID-19 infection side effects. ACE2 is also expressed in lymphocytes and following infection with the virus, their population decreases and the immune system weakens [13].

Also, different isoenzymes of CA have a high rate of expression in various tissues with critical biochemical functions in the pH homeostasis and alveolar O_2_-CO_2_ exchange [64]. After the inhalation, the oxygen is entered to alveoli of lungs with higher pressure than blood capillaries and delivered to hemoglobin in red blood cells (RBCs) [65]. Then, H_2_CO_3_ is catalyzed to CO_2_ and water molecule by CA ll of RBCs. In the acute COVID-19, following the destructive effects of virus on the pulmonary cells, the O_2_-CO_2_ exchange rate is impaired, leading to respiratory acidosis and shifting the pH of blood to acidic state and also increasing the concentration of blood CO_2_ [66]. Subsequently, the kidney proximal tubular cells increase reabsorption of HCO_3_^−^ leading to acid and ammonium excretion [67]. Then, H^+^ of the tubular fluid is combined with HCO_3_^−^ producing H_2_CO_3_, which is catalyzed to H_2_O and CO_2_ by CAs localized in kidney tissue [68]. Hence, as the consequence of infection of various cells including pulmonary cells of lungs, myocytes of heart, proximal tubular cells of kidney, and other ACE2-expressing cells (which mainly express CAs) with SARS-CoV-2 and reducing the ability of CO_2_ exhalation, the concentration of blood CO_2_ is elevated so even the lungs alveoli became unable in O_2_-CO_2_ exchange with pulmonary artery and vein through the CA enzymatic reaction [69]. The respiratory acidosis is associated with weakness of the respiratory muscles, arrhythmias, hypotension, myocardial depression, and decreasing in renal blood flow [66]. As the immunological aspect, the respiratory acidosis induces apoptosis and release of proinflammatory cytokines following RAS activation and raising the expression level of angiotensinogen by leukocytes [70]. Finally, the respiratory acidosis can lead to pulmonary edema, severe hypoxia as well as heart and renal failures [66]. Regarding to coexpression analyses results and referring to lack of sufficient information related to human CA VB, we couldn’t find the reason of the coexpression prediction of neprilysin and ACE2 with CA VB, which needs further experiments in future.

## Conclusions

Overally, due to high expression rate of ACE2 in the human organs and presence of a potential association with neprilysin in RAS and CAs, SARS-CoV-2 could be considered as an aggressive pathogen to all human organs which express ACE2. Therefore, this pathologic association (ACE2-Neprilysin-CA) complex can present valuable physiologic information to clinicians to innovate more influential treatment protocols against COVID-19.

## Data Availability

All data analyzed in this study were prepared from online databases, which were included in this article.

## Abbreviations

ACE2: Angiotensin-converting enzyme 2
ARDS: Acute respiratory distress syndrome
HCO_3_^−^: Bicarbonate
H_2_CO_3_: Carbonic acid
CA: Carbonic anhydrase
COVID-19: Coronavirus disease 2019
CO_2_: Carbon dioxide
CRP: C-reactive protein
DC-SIGN: Dendritic cell-specific intercellular adhesion molecule-3-grabbing non-integrin
E: Envelope
HBOT: Hyperbaric oxygen therapy
HIV: Human immunodeficiency virus
KEGG: Kyoto Encyclopedia of Genes and Genomes
M: Membrane
MME: Membrane metallo-endopeptidase
MrgprD: Mas-related G protein-coupled receptor
N: Nucleocapsid
O_2_: Oxygen
RAS: Renin angiotensin system
RBC: Red blood cell
S: Spike
SARS-CoV-2: Severe acute respiratory syndrome coronavirus 2
